# EZH2 Dynamically Associates With Non-coding RNAs in Mouse Hearts After Acute Angiotensin II Treatment

**DOI:** 10.3389/fcvm.2021.585691

**Published:** 2021-02-25

**Authors:** Shun Wang, Ningning Guo, Shuangling Li, Yuan He, Di Zheng, Lili Li, Zhihua Wang

**Affiliations:** ^1^Department of Cardiology, Renmin Hospital of Wuhan University, Wuhan, China; ^2^Central Laboratory, Renmin Hospital of Wuhan University, Wuhan, China

**Keywords:** epigenetics, EZH2, long non-coding RNAs, small nucleolar RNAs, RIP-Seq

## Abstract

Enhancer of zeste 2 (EZH2) governs gene reprogramming during cardiac hypertrophy through epigenetic remodeling, a process regulated by numerous non-coding RNAs (ncRNAs). However, the dynamic interaction between EZH2 and ncRNAs upon hypertrophic stimulation remains elusive. Here we performed an unbiased profiling for EZH2-associated ncRNAs in mouse hearts treated with Angiotensin II (AngII) at different time points (0, 4, and 24 h). The interactions between EZH2 and long ncRNAs (lncRNAs), Chaer, Mirt1, Hotair, and H19, were validated by PCR. RIP-seq analysis identified a total of 126 ncRNAs to be significantly associated with EZH2. These ncRNAs covers all five categories including intergenic, antisense, intron-related, promoter-related and both antisense and promoter-related. According to their changing patterns after AngII treatment, these ncRNAs were clustered into four groups, constantly enhanced, transiently enhanced, constantly suppressed and transiently suppressed. Structural prediction showed that EZH2 bound to hairpin motifs in ncRNAs including snoRNAs. Interaction strength prediction and RNA pull-down assay confirmed the direct interaction between EZH2 and Snora33. Interestingly, two antisense lncRNAs of Malat1, Gm20417, and Gm37376, displayed different binding patterns from their host gene after AngII treatment, suggesting a crucial role of this genomic locus in modulating EZH2 behavior. Our findings reveal the profile of EZH2-associated ncRNAs upon hypertrophic stimulation, and imply a dynamic regulation of EZH2 function in cardiac hypertrophy.

## Introduction

In mammalians, only 1–2% of the genome is responsible for protein coding though 70–90% is transcriptionally active (1, 2). The vast majority of human DNA are transcribed into non-coding RNAs (ncRNAs) including long non-coding RNAs (lncRNAs) (2, 3). LncRNAs differ from short ncRNAs simply in length with 200-nt as the separatrix. Although once considered as junk RNAs, ncRNAs have recently been recognized as crucial regulatory molecules involved in a series of cellular processes, such as chromatin remodeling, genomic stability, transcription, post-transcriptional modifications and signal transduction (4, 5). There is an increasing interest to explore the role of ncRNAs in numerous human diseases including cardiovascular diseases.

The function of ncRNAs largely depends on their subcellular location (6). A number of nucleus-localizing lncRNAs have been found directly interacting with epigenetic modifiers and modulate gene expression in *cis* or *trans* manners (6–10). Enhancer of zeste 2 (EZH2), a subunit of polycomb repressive complex 2 (PRC2) catalyzing tri-methylation of histone H3 at lysine 27 (H3K27me3) (11, 12), represents a major target of lncRNAs inside nucleus (13–18). Some lncRNAs function as a decoy by interacting with EZH2 to prevent PRC2 from binding with chromatin or to interfere with the allosteric activation of PRC2, without impeding its methylation catalytic activity (19, 20). Whereas, lncRNA-p21 has been proposed to disrupt the PRC2 complex and promote the binding of EZH2 with genes independent of the PRC2 complex, which triggers methylation of targeted genes (21, 22).

Cardiac hypertrophy is a common pathological process of many types of cardiovascular diseases (23). A number of lncRNAs, such as maternally expressed 3 (Meg3), myosin heavy-chain-associated RNA transcripts (Mhrt), cardiac hypertrophy-related factor (Chrf), Myocardial Infarction–Associated Transcript (Miat) have been reported to be involved in cardiac hypertrophy (24–27). We previously identified a heart-enriched lncRNA cardiac hypertrophy associated epigenetic regulator (Chaer), which transiently interacts with EZH2 at early phase of cardiac hypertrophy induced by transaortic constriction (TAC) surgery and prevents its targeting to the promoter region of hypertrophic genes (28), implicating a highly dynamic regulation of EZH2 by lncRNAs. However, the molecular mechanism for this dynamic process has yet been clarified.

In a previous study, we characterized the tissue-specificity of EZH2-associated lncRNAs using RNA immuno-precipitation coupled with sequencing (RIP-seq) (29). Here we performed an unbiased profiling for EZH2-binding ncRNAs in mouse hearts following Angiotensin II (AngII) treatment. Our findings reveal the landscape of EZH2-associated ncRNAs during early cardiac hypertrophy and provide novel insights into the dynamics of EZH2-mediated epigenetic remodeling.

## Materials and Methods

### Animals

Male mice aged 10 weeks were housed in specific-pathogen-free (SPF) conditions with controlled temperature, humidity, and light and free access to food and water. Animal experiments were performed conforming to the 8th Edition of the Guide for the Care and Use of Laboratory Animals (Guide NRC, 2011) published by the US National Institutes of Health. Mice were randomly assigned to Sham, Angiotensin II (AngII) 4 h and AngII 24 h groups with two mice for each group. An osmotic minipump (Alzet, Cupertino, CA, USA) was implanted subcutaneously to deliver AngII (Sigma, St. Louis, MO) at a rate of 1 mg/kg/min as previously described (30). After infusion for 4 or 24 h, mice were sacrificed by dislocation of infra-cervical spine. Left ventricles were the quickly separated, washed in PBS and frozen in liquid nitrogen until use. Saline was infused instead of AngII for Sham groups.

### Cell Culture and Plasmids

Mouse embryonic fibroblasts (MEFs) were maintained in DMEM supplemented with 10% fetal bovine serum, 2 mM L-glutamine, 100 U/mL penicillin and 100 μg/ml streptomycin. Gm20417 and Gm37376 were clone from mouse heart cDNA library into pcDNA3.1-CMV expression plasmid, and transfected into MEFs using Lipofectamine 2000 (Invitrogen, Thermo Fisher Scientific Inc.). Cells were harvested 24 h after transfection.

### Western Blot

Frozen heart tissues or cells were homogenized in protein lysis buffer (50 mM HEPES [pH7.4], 150 mM NaCl, 1% Triton X-100, 1 mM EDTA, 1 mM EGTA, 1 mM glycerophosphate, 2.5 mM sodium pyrophosphate, 1 mM Na3VO4, 20 mM NaF, 1 mM phenylmethylsulfonyl fluoride, 1 mM DTT, 1 × complete protease inhibitor tablet [Roche, Swiss]). Total lysates were separated on 10 or 12% Bis-Tris gels and transferred onto PVDF membranes (Millipore). The blots were probed with antibodies for H3 (#12648), H3K27me3 (#9733), EZH2 (#5246), and GAPDH (#5174) from Cell Signaling Technologies, Inc. Protein signals were detected using HRP conjugated secondary antibodies and enhanced chemiluminescence (ECL) western blotting detection regents (Thermo Fisher Scientific, MA, USA).

### RNA Immune-Precipitation

RNA immune-precipitation (RIP) was performed essentially as previously described (28, 29, 31). Left ventricles weighting ~100 mg from each group were homogenized in 500 μl of polysome lysis buffer (10 mM HEPES-KOH [pH 7.0], 100 mM KCl, 5 mM MgCl_2_, 25 mM EDTA, 0.5% IGEPAL, 2 mM dithiothreitol [DTT], 0.2 mg/mL Heparin, 50 U/mL RNase OUT [Life Technologies], 50 U/mL Superase IN [Ambion] and 1 × complete protease inhibitor tablet [Roche]). The suspension was centrifuged at 14,000 *g* at 4°C for 10 min to remove debris. Lysates were incubated with 500 ng normal IgG (Cell Signaling Technologies, MA, USA; #2729, 1:200) or anti-EZH2 (Cell Signaling Technologies; #5246, 1:200) at 4°C overnight on an inverse rotator. Protein A-sepharose beads (Life Technologies, 50 μl per tube) were first blocked in NT2 buffer (50 mM Tris-HCl (pH 7.5), 150 mM NaCl, 1 mM MgCl_2_, and 0.05% IGEPAL) supplemented with 5% BSA, 0.02% sodium azide and 0.02 mg/mL heparin at 4°C for 1 h, and then added into the lysates followed by a 3-h incubation at 4°C on an inverse rotator. The beads were subsequently washed five times in NT2 buffer. RNAs were released by incubating in proteinase K buffer (50 mM Tris (pH 8.0), 100 mM NaCl, 10 mM EDTA, 1% SDS, and 1 U/mL proteinase K) for 30 min at 65°C, pelleted by adding an equal volume of isopropanol and centrifuged at 12,000 *g* at 4°C for 10 min. RNAs were washed once with 75% ethanol and stored at −80°C until use.

### RNA Pull-Down Assay

To validate the direct interaction between EZH2 and Snora33, we employed the tagged RNA pull-down assay adjusted from a previous report (32). Snora33 was obtained using an T7 *in vitro* transcription kit (Merk Inc.), followed by biotin conjugation using the Biotinylation Kit (Thermo Fisher Scientific Inc.). Biotinylated Snora33 was then incubated with MEF lysates in SA-RNP lysis buffer (20 mM Tris-HCl [pH7.5], 150 mM NaCl, 1.5 mM MgCl_2_, 2 mM DTT, 50 U/ml RNase OUT [Invitrogen], 50 U/ml Superase IN [Ambion], 1× complete protease inhibitor tablet [Roche]) for 4 h at 4°C. Streptavidin sepharose beads were blocked with 500 ng/μl yeast tRNA and 1 mg/ml BSA in SA-RNP lysis buffer before added into cell lysates and incubated at 4°C for 2 h on a rotator. The beads were then pelleted and washed for 5 times with SA-RNP washing buffer (20 mM Tris-HCl [pH7.5], 300 mM NaCl, 5 mM MgCl_2_, 2 mM DTT, 50 U/ml RNase OUT [Invitrogen], 50 U/ml Superase IN [Ambion], 1× complete protease inhibitor tablet [Roche]). After the last wash, RNA-bound proteins were eluted by addition of 5% RNase A (NEB) in low salt buffer (20 mM Tris-HCl [pH7.5], 30 mM NaCl, 5 mM MgCl2, 2 mM DTT, 1× complete protease inhibitor tablet [Roche]) for 30 min at 4°C. The eluted proteins, together with the flow-through and input samples, were then boiled in 4× LDS sample buffer (Life Technologies) and used for immunoblot analysis.

### RNA Sequencing

Two independent RIP products from each group were mixed before reverse transcription into cDNA sequencing library using KAPA Stranded RNA-Seq Library Preparation Kit. The libraries were subjected to quality validation using the Agilent Bioanalyzer 2100, and sequenced using Illumina NextSeq 500 in DNA Link USA Inc. The reads were mapped to mouse genome (mm10) using TopHat2 (28), and visualized on the UCSC browser (http://genome.ucsc.edu). LncRNAs were picked out according to NONCODE database (33). Screening criteria was set as reads >1.0; ratio of anti-EZH2 group relative to normal IgG group >2 in at least one tissue.

### Real-Time PCR

Briefly, 1 μg RNA was reverse-transcribed into first-strand cDNA using the Superscript III first-strand synthesis kit (Life Technologies, NY, USA) with random primers. Real-time PCR was performed using the CFX96 Real-Time PCR Detection System (Bio-Rad, CA, USA) using the iQ SYBR Green Supermix (Bio-Rad). Values were normalized to IgG controls. Primers are listed below.

Chaer: F-5′-TCCAATGAGGGAAGCGAAGC-3′, R-5′-GTCCGATGCCAGTTCCAGTT-3′;

Hotair: F-5′-CTTTCAAGGCCTGTCTCCTG-3′, R-5′-CAACATTCTAGCTGCACGGA-3′;

H19: F-5′-AGACTAGGCCAGGTCTCCAG-3′, R-5′-TAGAGGCTTGGCTCCAGGAT-3′;

Mirt1: F-5′-TGGGAGGCTGAGGCTAAGAT-3′, R-5′-ACCTACCCCTACTGCTGGAG-3′.

### In Silicon RNA Secondary Structure Prediction

RNA secondary structure was predicted by RNAfold WebServer (http://rna.tbi.univie.ac.at/cgi-bin/RNAfold.cgi) based on minimum free energy (MFE) and partition function. The interaction strength between EZH2 and ncRNAs were predicted by an online program using random RNA sequences as (http://s.tartaglialab.com/page/catrapid_group) (34).

### Statistics

Data were presented as mean ± SEM for repeated analyses. Statistical analysis was performed by one-way ANOVA and Turkey HSD *post hoc* test using GraphPad Prism. Comparisons between two groups were analyzed by student's *t*-test. *P* < 0.05 was considered statistically significant. EZH2 RIP data were presented as mean ± s.d. from two independent RIP products.

## Results

### Validation of the RIP Method

**Figure 1:**
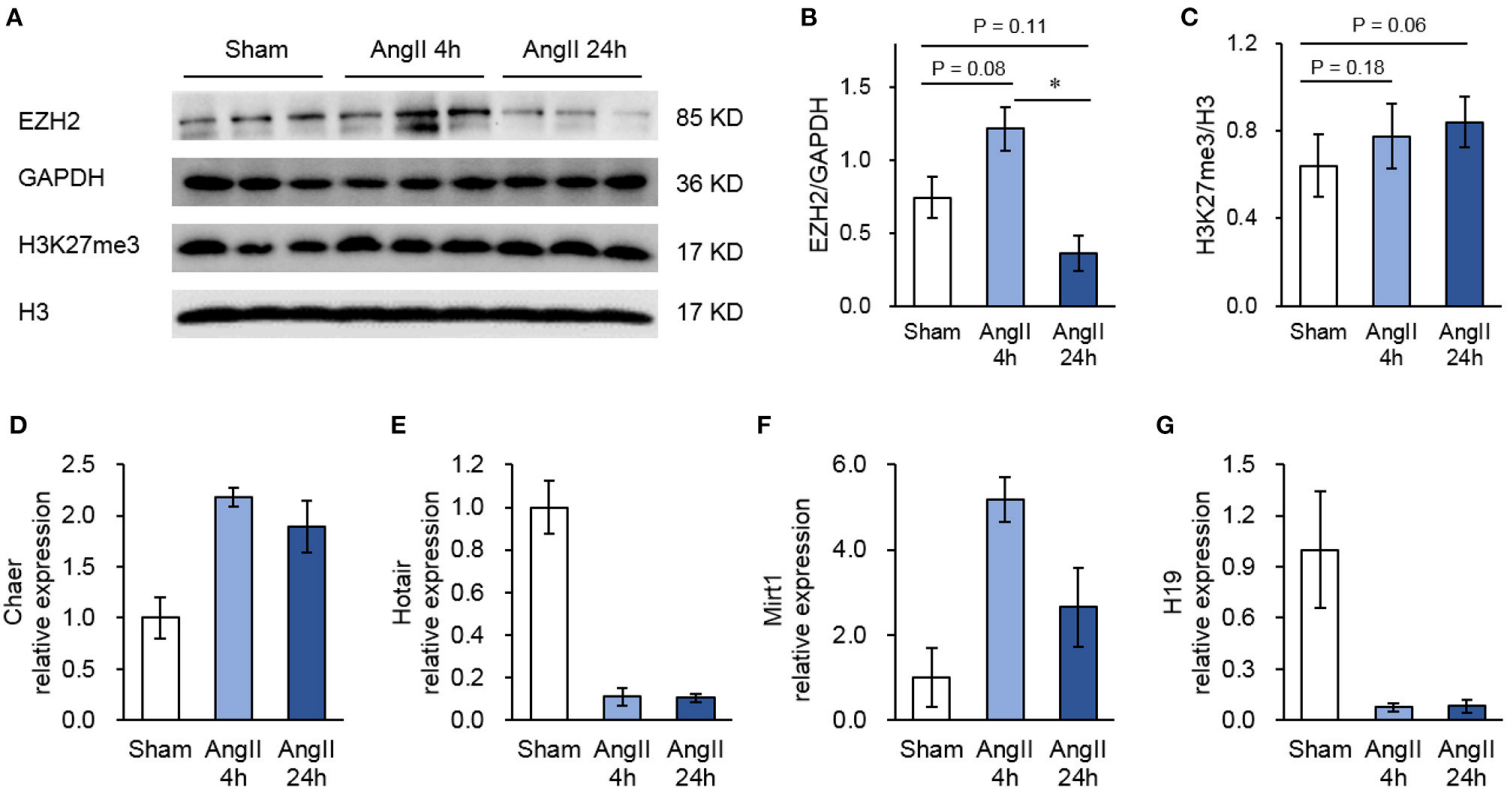
Validation of the RIP method to pull-down Ezh2-binding lncRNAs. **(A)** Representative immunoblots showing the expression of EZH2, GAPDH, H3K27me3, and H3 in mouse hearts treated with Angiotensin II (AngII) at 0 (Sham), 4 and 24 h. **(B,C)** Analyzed results of EZH2 expression in relative to GAPDH **(B)** and H3K27me3 expression in relative to H3 **(C)**. Data are mean ± SEM; *P < 0.05 vs. sham; *n* = 3. **(D–G)** Relative expression of known EZH2-binding lncRNAs including Chaer **(D)**, Hotair **(E)**, Mirt1 **(F)**, and H19 **(G)** in mouse hearts treated with Angiotensin II (AngII) at 0 (Sham), 4 and 24 h. Data are mean ± SD from two independent experiments.

Though not reaching statistical significance, AngII administration upregulated the protein expression of EZH2 after 4 h but reduced it after 24 h (Figures 1A,B). This pattern was inconsistent with the changes of global H3K27me3 (Figures 1A,C), suggesting a regulation of EZH2 activity by other factors. To explore the EZH2-associated ncRNAs, we then performed native RIP analysis followed by sequencing. After pull-down using anti-EZH2 or normal IgG antibodies, we firstly validated the RIP procedure with known EZH2-binding lncRNAs, including cardiac hypertrophy associated epigenetics regulator (Chaer), HOX transcript antisense RNA (Hotair), myocardial infarction-associated transcript 1 (Mirt1), and H19 (28, 35–37) using real-time PCR. The results showed that Chaer-EZH2 interaction was quickly enhanced after AngII treatment, whereas Hotair-EZH2 interaction was repressed (Figures 1D,E), which is consistent with our previous findings in cardiomyocyte hypertrophy induced by phenylephrine (28). In addition, Mirt1 and H19 were also detected in the EZH2 interactome with the former being enhanced while the latter being suppressed after AngII treatment (Figures 1F,G). These data verify the success of our RIP procedure, as well as the pro-hypertrophic effect of AngII treatment.

### Identification of EZH2-Associated ncRNAs

**Figure 2:**
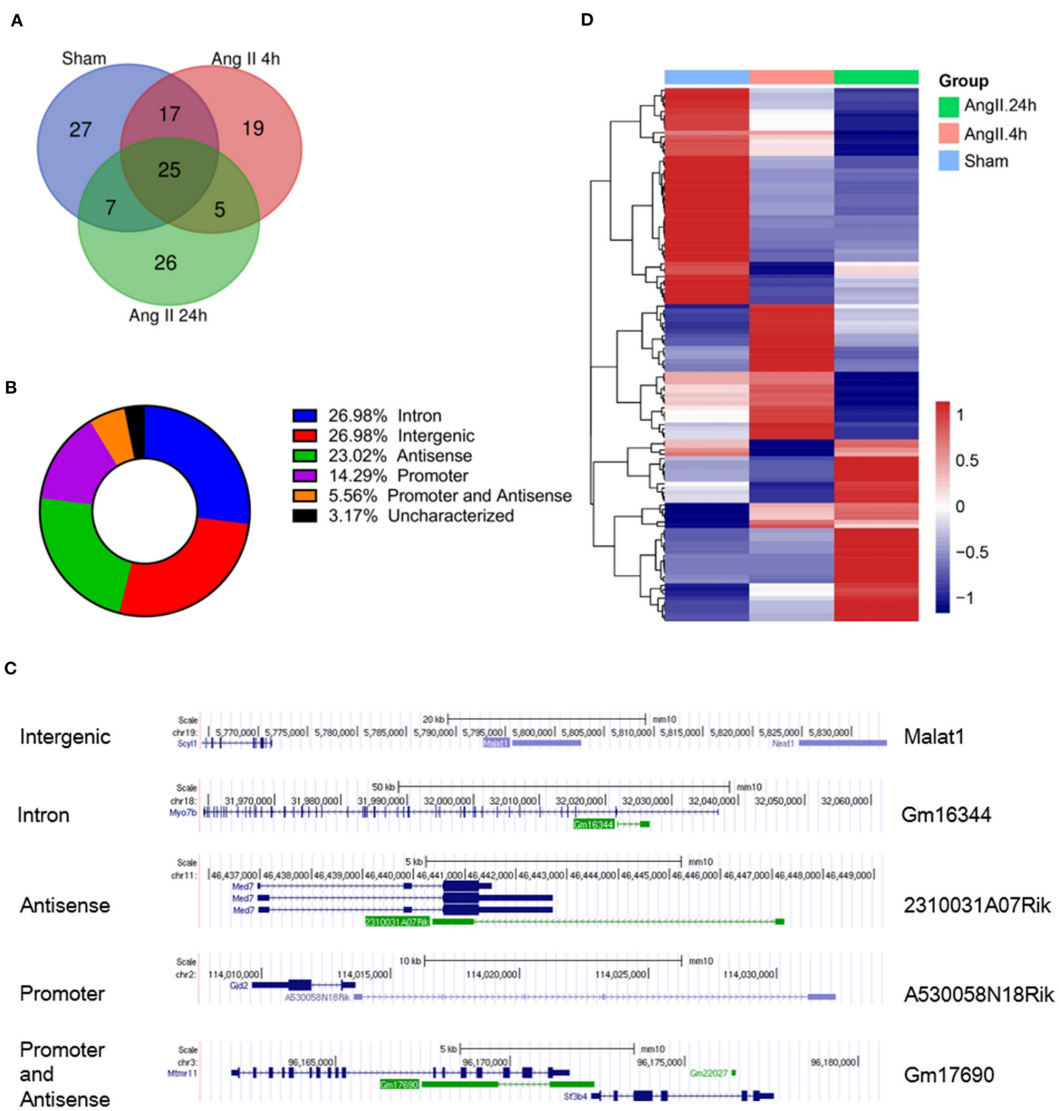
Profiles of EZH2-binding ncRNAs in mouse hearts treated with AngII at different time points. **(A)** Venn diagram showing the number of overlapped ncRNAs from three groups as indicated. **(B)** Pie chart showing the distribution of ncRNA categories according to their genomic features. **(C)** Representative ncRNA for each category. **(D)** Heat map showing hierarchical clustering of changed EZH2-associated ncRNAs after AngII treatment at different time points. Up-regulated and down-regulated genes are colored in blue and yellow, respectively.

In the RIP-seq analysis, we identified a total of 126 ncRNAs associated with EZH2 in all groups with no 0 reads in any group and over 2-fold enrichment in anti-EZH2 relative to normal IgG (Figure 2A). Separately, there were 76 ncRNAs in the Sham group, 66 in the 4-h AngII group and 63 in the 24-h AngII group. Among these EZH2-associated ncRNAs, 25 (20%) were shared by all three groups (Figure 2A and Table 1). From their genomic locations, these ncRNAs covered all five categories, including intergenic, antisense, intron-related, promoter-related and both antisense- and promoter-related (Figures 2B,C), suggesting a vast functional diversity of EZH2 in both local and global epigenetic regulations. Clustering analysis showed that EZH2-associated lncRNAs from AngII 4 h and AngII 24 h groups were clustered together, apart from that from the Sham group (Figure 2D), suggesting a quick response of EZH2-mediated epigenetic reprogramming to AngII stimulation whereby lncRNAs are functionally involved.

**Table 1:** Shared EZH2-associated lncRNAs in all groups.

**Gene name**	**Tracking ID**	**Ratio to IgG**			**Category**
		**Sham**	**AngII 4 h**	**AngII 24 h**	
Gas5	XLOC_001290	916.469	20.043	6.252	Intergenic
2410002F23Rik	XLOC_033560	78.164	835.468	1,041.137	Intergenic
Gm29055	XLOC_000696	17.072	7.540	8.856	Intron
Gm28268	XLOC_040459	12.568	3.984	2.580	Intergenic
Gm3052	XLOC_001918	10.709	2.320	4.242	Intergenic
Gm17131	XLOC_028763	9.803	4.295	2.793	Antisense
Gm9917	XLOC_039729	9.625	6.481	18.211	Promoter and antisense
Gm17132	XLOC_028762	9.357	9.789	6.614	Antisense
Gm37954	XLOC_002666	9.327	4.182	10.852	Intron
Gm26905	XLOC_036601	9.043	7.215	8.242	Antisense
Gm21897	XLOC_013754	7.216	4.441	3.814	Intergenic
Gm13722	XLOC_020816	5.984	4.678	2.653	Intergenic
Gm16793	XLOC_037281	5.346	2.848	16.772	Promoter
Gm37515	XLOC_023239	4.846	7.710	10.978	Intergenic
Gm20417	XLOC_017336	4.806	3.038	4.549	Antisense
C030037D09Rik	XLOC_005812	3.961	3.766	2.246	Promoter
Gm37376	XLOC_017337	3.838	5.611	10.619	Antisense
Gm37086	XLOC_001530	3.393	5.279	4.102	Intergenic
Gm26869	XLOC_021846	2.920	2.364	3.833	Antisense
Gm16344	XLOC_016557	2.904	5.573	3.629	Antisense
Bvht	XLOC_016764	2.851	2.056	2.034	Intergenic
Malat1	XLOC_017847	2.767	3.784	3.065	Intergenic
5330426P16Rik	XLOC_014583	2.758	2.656	3.264	Uncharaterized
Gm15662	XLOC_003933	2.046	3.031	2.679	Antisense
Gm17690	XLOC_024078	2.004	9.052	2.132	Promoter and antisense

### Dynamic Association Between EZH2 and ncRNAs During AngII Stimulation

**Figure 3:**
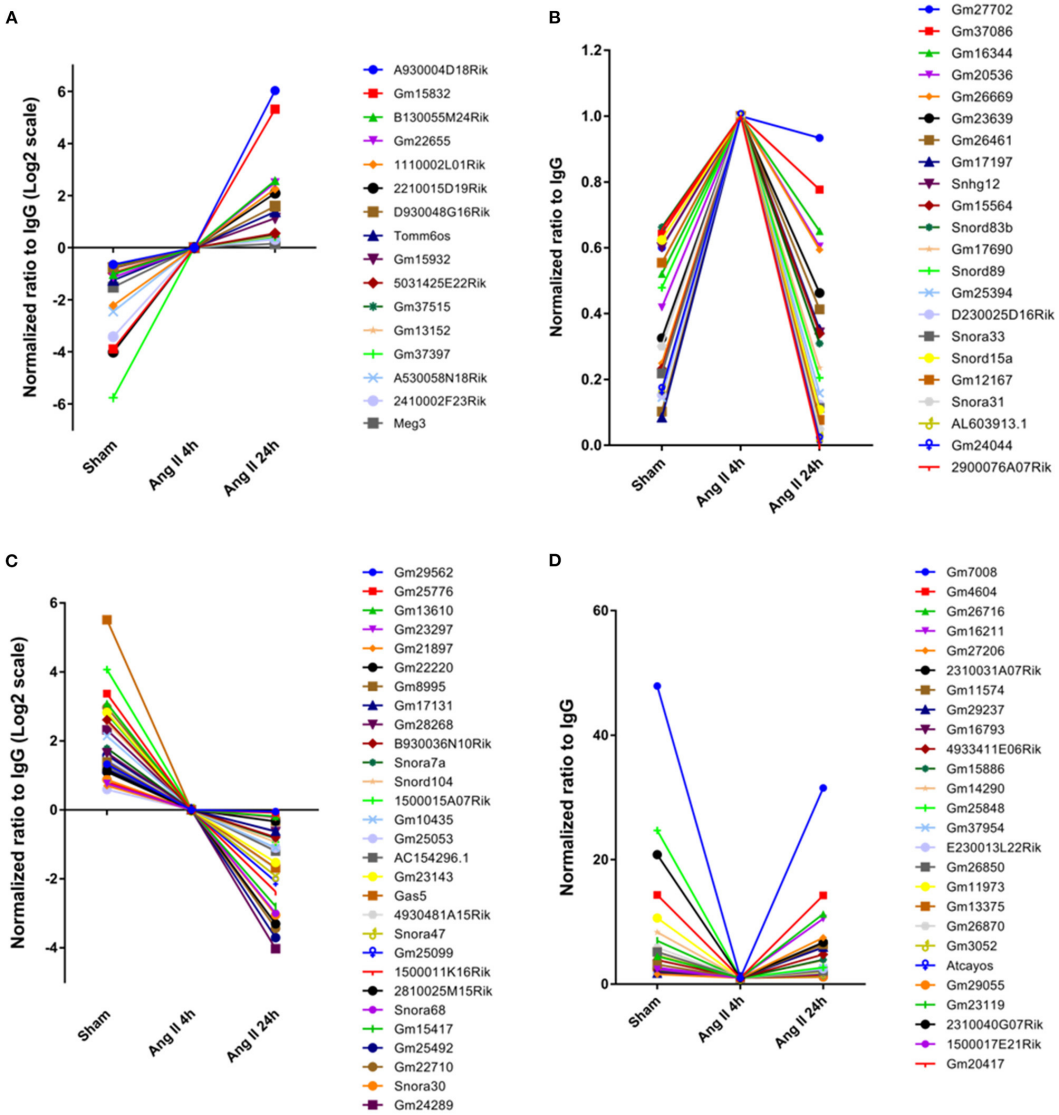
Stratification of EZH2-associated ncRNAs according to their changing pattern after AngII treatment. **(A)** Constantly enhanced EZH2-binding ncRNAs in both AngII 4 and 24 h groups. **(B)** Transiently enhanced EZH2-binding ncRNAs after 4 h of AngII treatment but attenuated after 24 h of AngII treatment. **(C)** Constantly suppressed EZH2-binding ncRNAs in both AngII 4 and 24 h groups. **(D)** Transiently suppressed EZH2-binding ncRNAs after 4 h of AngII treatment but reversed after 24 h of AngII treatment. Data were normalized to the AngII 4 h group.

We further analyzed the impact of AngII on EZH2-ncRNA interaction. A total of 93 EZH2-associated ncRNAs were altered >1.5-folds in all three comparisons. According to their alteration pattern following AngII treatment, these ncRNAs were classified into four groups; i.e., constantly enhanced (Figure 3A), transiently enhanced (Figure 3B), constantly suppressed (Figure 3C) and transiently suppressed (Figure 3D). Several known EZH2-interacting lncRNAs appeared in the list (37–40); e.g., growth arrest specific 5 (Gas5) was dissociated from EZH2 while maternally expressed 3 (Meg3) was recruited after AngII administration (Figures 3A,C). These results suggest that the interaction between EZh2 and ncRNAs is a highly dynamic process and may contribute to the epigenetic remodeling during early cardiac hypertrophy.

### Structural Characteristics of EZH2-Associated ncRNAs

**Figure 4:**
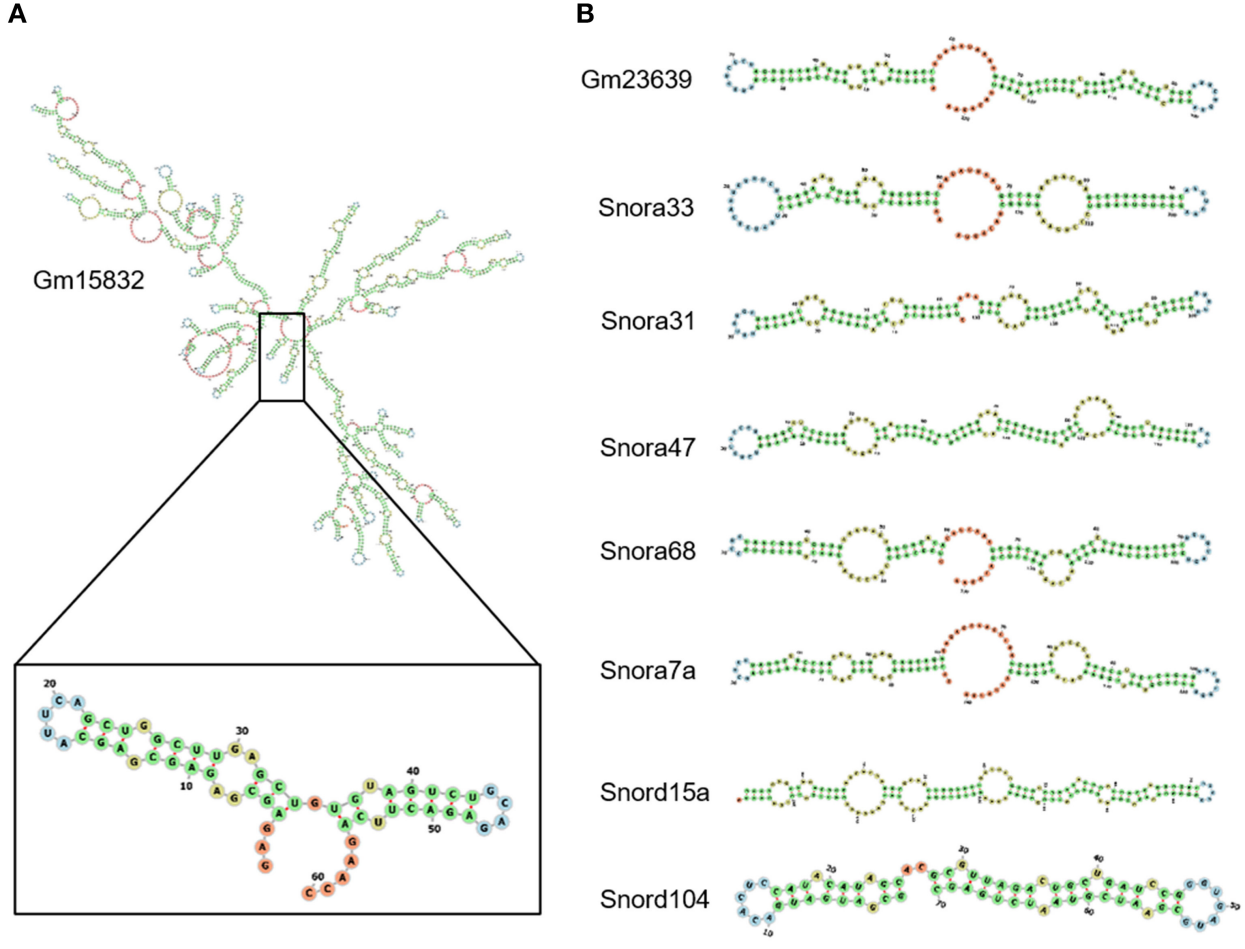
Structural characteristics of EZH2-associated ncRNAs. Predicted secondary structure of lncRNA Gm15832 **(A)**, Snora family ncRNAs and Snord family ncRNAs **(B)** using RNAfolder.

It has been reported that EZH2 tends to bind RNA motifs with tandem tetra-loop hairpins (28). We then analyzed the secondary structure of candidate ncRNAs using RNAfold (25). In Gm15832, a constantly enhanced EZH2-associated lncRNA during AngII treatment, we identified a potential 60-mer motif (100–159 nt) with a similar structure as that from Chaer or Hotair (28) (Figure 4A). Interestingly, EZH2 bound to a number of small nucleolar RNAs (snoRNAs) containing the H/ACA box, including Snora7a, Snora31, Snora33, Snora47, and Snora68 (Figure 4B), suggesting a role of EZH2 in rRNA processing (41, 42). Moreover, snoRNAs containing the C/D box, like Snord15a and Snord104, were also detected in the EZH2 interactome (Figure 4B). A number of uncharacterized ncRNAs (e.g., Gm23639) displayed the typical structural feature as snoRNAs (Figure 4B).

**Figure 5:**
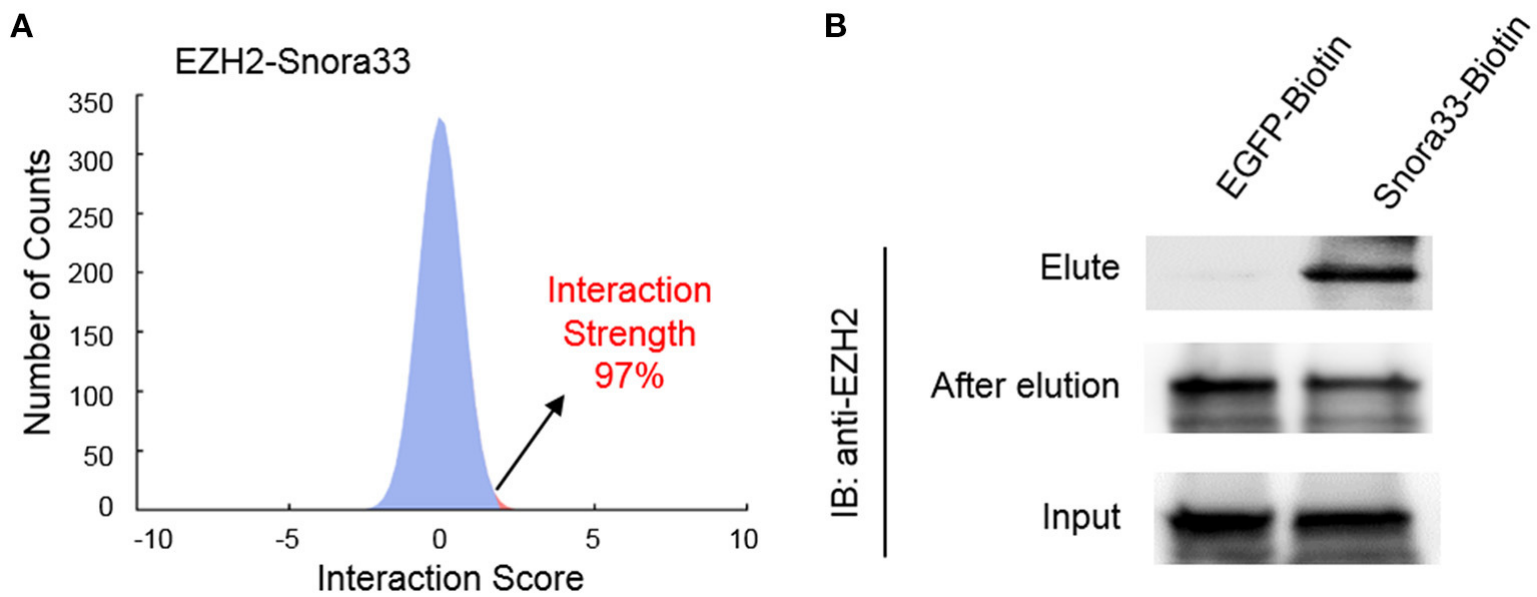
Validation of the interaction between EZH2 and Snora33. **(A)** Predicted interaction strength between EZH2 and Snora33 using catRAPID. Among a thousand of random RNA sequences, Snora33 ranked 97% propensity to interact with EZH2. **(B)** RNA pull-down analysis to confirm the direct interaction between Snora33 and EZH2. Biotin was conjugated to the 3′ of Snora33 RNA *in vitro*, followed by streptavidin pull-down assay. Immunoblotting was performed with anti-EZH2 antibody using the elute, flow-through and input samples.

To validate the interactions, we performed an online prediction for the interaction strength using catRAPID algorithm (34). The results showed that Snora33 had the highest interaction strength (97%) with EZH2 protein among others (Figure 5A). RNA pull-down assay showed that the biotin-tagged Snora33 could strikingly pull down EZH2 compared with the EGFP control, along with a decrease of endogenous EZH2 in the flow-through sample (Figure 5B). These data further validate the validity of the EZH2-binding ncRNAs.

### Malat1 Genomic Locus Is Involved in AngII-Induced Epigenetic Reprogramming

**Figure 6:**
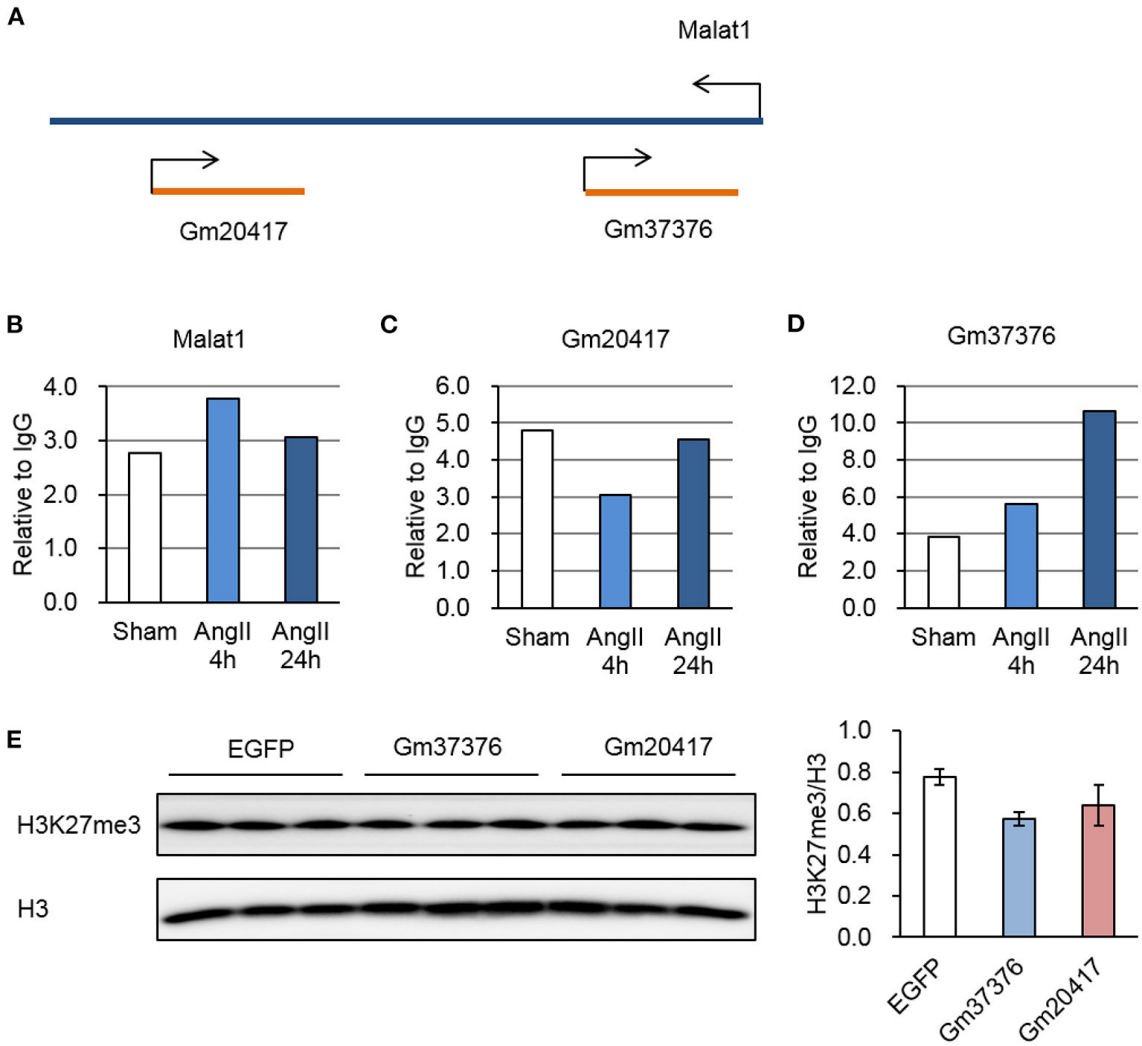
Dynamic interaction between EZH2 and Malat1 locus-derived lncRNAs during AngII treatment. **(A)** Schematic diagram of the Malat1 genomic structure together with two antisense lncRNAs, Gm20417, and Gm37376. **(B–D)** Relative reads of Malat1 **(B)**, Gm20417 **(C)** and Gm37376 **(D)** normalized to corresponding IgG groups. **(E)** Impact of Gm37376 and Gm20417 on H3K27me3 in MEFs. Cells were collected 24 h after transfection, and the lysates were immunoblotted with anti-H3K27me3 and anti-H3 antibodies. Data are mean ± SEM; *n* = 3.

We previously identified two antisense lncRNAs, Gm20417, and Gm37376, at the gene locus of Malat1 displaying high tissue specificity (29). Here we found that all of these three lncRNAs significantly associated with EZH2; however, whereas Malat1-EZH2 interaction was transiently enhanced after AngII treatment, Gm20417-EZH2 interaction was transiently suppressed and Gm37376-EZH2 interaction was constantly enhanced (Figures 6A–D), suggesting a complex regulation of EZH2 function in AngII-induced cardiac hypertrophy. We then cloned Gm20417 and Gm37376, and expressed them in MEFs. Western blot analysis showed that Gm37376 and Gm20417 did not significantly alter the global level of H3K27me3 (Figure 6E), suggesting a regional regulation rather than regulating the global activity of EZH2 by these ncRNAs. These data also implicate that the Malat1 gene is a crucial locus to modify EZH2 function under stress.

## Discussion

Accumulating evidence highlights the crucial role of ncRNAs in cardiac hypertrophy. In this study, we found that EZH2 serves as a major target for not only lncRNAs but also snoRNAs. In addition to validating known EZH2-binding ncRNAs (e.g., Gas5, Meg3, and Malat1), our findings reveal a large panel of novel ncRNAs involved in the dynamic regulation of the EZH2 function during AngII-induced cardiac hypertrophy.

The dynamic interaction between EZH2 and ncRNAs suggests a complex role of EZH2 in the pathogenesis of cardiac hypertrophy. A huge change was observed as early as 4 h after AngII treatment. This is consistent with our previous report that Chaer-EZH2 interaction was transiently enhanced at the early phase of cardiac hypertrophy and that overall H3K27me3 was quickly diminished upon hypertrophic stimulation (28). The shift in binding property might rely on certain post-translation modifications of the EZH2 protein, since it can be modified by phosphorylation or glycosylation (43, 44). However, we did not detect changes of known EZH2 phosphorylation at T345 and T487 at the early phase of cardiac hypertrophy, though they were indeed sensitive to mTOR (mechanistic target of rapamycin) inhibition (Data not shown), implicating that other modifications or intrinsic EZH2 gene heterogeneity might underlie its dynamic interaction with ncRNAs. Further investigation is required to address this question in more details.

The EZH2-associated ncRNAs display diverse genomic features including intergenic, antisense, intron-related, promoter-related and both antisense- and promoter-related, suggesting diverse epigenetic regulations both *in cis* or *in trans*. EZH2 has been reported to undergo specificity and dynamics regulations in the context of heart development, whereby EZH2 is responsible for catalyzing H3K27me3 at the bivalent promoters of developmental genes (45–47). In Ezh2-deficient adult hearts, fetal genes are upregulated, giving rise to cardiac hypertrophy (48, 49). Regulatory RNAs are essential for PRC2 chromatin occupancy and function (50). Our previous study revealed that lncRNA Chaer transiently interacted with EZH2 at the early phase of cardiac hypertrophy. This interaction interfered with the binding of PRC2 to the promoters of pathological genes, thus allowing their activation by transcription factors (28). Here we found more ncRNAs displaying similar changing pattern during early hypertrophy, such as Gm24044, 2900076A07Rik, and Snora33 (Figure 2B). This mechanism is different from previously reported scaffold or guide ncRNAs like Hotair and Gtl2 (8, 37). Whether they also function like Chaer but not other ncRNAs needs further functional investigation. Along with the upregulated ncRNAs, the interaction between EZH2 and a number of ncRNAs, such as Gm7008 and Gm4604, was transiently suppressed upon AngII stimulus. These data suggest that ncRNAs compete for the access to the EZH2 binding site.

EZH2 does not contain a typical RNA-binding motif such as RRM (RNA recognition motif). Previous studies reveal that the residues 342–368 in full-length EZH2 constitute a RNA-binding domain (51). Long et al. (52) found that the N-terminal helix of EZH2 is the major RNA-binding site and has a preferential binding of G-quadruplex RNA. The structural characteristics of EZH2-binding RNA have not been fully elucidated so far. Our previously study has shown that Chaer is capable of binding EZH2 through a tandem tetra-loop motif, which shared with several other EZH2-binding ncRNAs (28). Among the dynamic EZH2- binding ncRNA after AngII treatment, we identified Gm15832 that has a similar motif recognized by EZH2 (Figure 4A). An interesting finding is that EZH2 bind a series of snoRNAs with a typical tandem stem-loop structure, which can be classified into C/D-box (SNORD) and H/ACA-box (SNORA) subfamilies (Figures 4B,C). Considering that snoRNAs mediate the rRNA acetylation and tRNA methylation modification (42), our findings indicate a potential role of EZH2 in mediating rRNA or tRNA maturation, as well as ribosome assembly inside nucleolus.

Although EZH2 has once been thought to promiscuously bind RNAs (53, 54), cumulating evidence including ours indicates that EZH2-binding ncRNAs possess specific structural commonness (28, 37). As found in RepA, Hotair and Chaer, a conserved tandem tetra-loop hairpin motif is responsible for the direct interaction between ncRNAs and EZH2 (28, 55). In this study, we screened the secondary structure for most of the identified ncRNAs. However, similar motif did not exist in all EZH2-associated ncRNAs except for Gm15832 (Figure 3A). Interestingly, EZH2-binding snoRNAs showed similar structural features (Figure 3B), suggesting divergent interaction patterns between EZH2 and ncRNAs possibly derived from different binding sites on the surface of EZH2 protein.

Compared with mRNAs, ncRNAs are less conserved among species (56). Similarly, most of the ncRNAs revealed in this study could not find their counterpart human homologs, but this does not exclude the possibility for certain structurally conserved counterparts to carry out the same function. One conserved lncRNA Malat1 was consistently detected in both the current and our previous reports (29). The role of Malat1 in cardiac hypertrophy is still under debate (57). The fact that three lncRNAs at this genomic locus could all bind to EZH2 and display diverse changing pattern after AngII treatment might add another layer to the Malat1-mediated epigenetic regulation. Moreover, novel function of EZH2 independent of forming PRC2 complex and subsequent H3K27me3 modification has been unveiled (58). It would be interesting to explore whether non-epigenetic function of EZH2 can be also modified by ncRNAs.

Taken together, our study provides direct evidence for the complex role of EZH2 during early cardiac hypertrophy, and unveils a novel access to modify EZH2 function through ncRNAs.

## Data Availability Statement

The RIP-seq data can be accessed in the GEO Database with accession number GSE167007.

## Ethics Statement

The animal study was reviewed and approved by Ethical Committee of Renmin Hospital of Wuhan University.

## Author Contributions

ZW designed and supervised the study. SW, NG, and SL performed the experiments and analyzed the data with input from YH, DZ, and LL. SL and ZW drafted the manuscript. All authors contributed to the article and approved the submitted version.

## Conflict of Interest

The authors declare that the research was conducted in the absence of any commercial or financial relationships that could be construed as a potential conflict of interest.
